# Fecal Bacteria as Non-Invasive Biomarkers for Colorectal Adenocarcinoma

**DOI:** 10.3389/fonc.2021.664321

**Published:** 2021-08-10

**Authors:** Biao Yuan, Bin Ma, Jing Yu, Qingkai Meng, Tao Du, Hongyi Li, Yueyan Zhu, Zikui Sun, Siping Ma, Chun Song

**Affiliations:** ^1^Department of Gastroenterological Surgery, Shanghai East Hospital, Tongji University of Medicine, Shanghai, China; ^2^Department of Colorectal Surgery, Cancer Hospital of China Medical University, Liaoning Cancer Hospital and Institute, Shenyang, China; ^3^Research and Development Department, Shanghai Personal Biotechnology Co., Ltd, Shanghai, China; ^4^ECNU-PERSONAL Joint Laboratory of Genetic Detection and Application, Shanghai Personal Biotechnology Co., Ltd, Shanghai, China

**Keywords:** colorectal adenocarcinoma, non-invasive diagnosis, biomarker, gut microbiome, 16s rRNA sequencing, machine learning

## Abstract

Colorectal adenocarcinoma (CRC) ranks one of the five most lethal malignant tumors both in China and worldwide. Early diagnosis and treatment of CRC could substantially increase the survival rate. Emerging evidence has revealed the importance of gut microbiome on CRC, thus fecal microbial community could be termed as a potential screen for non-invasive diagnosis. Importantly, few numbers of bacteria genus as non-invasive biomarkers with high sensitivity and specificity causing less cost would be benefitted more in clinical compared with the whole microbial community analysis. Here we analyzed the gut microbiome between CRC patients and healthy people using 16s rRNA sequencing showing the divergence of microbial composition between case and control. Furthermore, ExtraTrees classifier was performed for the classification of CRC gut microbiome and heathy control, and 13 bacteria were screened as biomarkers for CRC. In addition, 13 biomarkers including 12 bacteria genera and FOBT showed an outstanding sensitivity and specificity for discrimination of CRC patients from healthy controls. This method could be used as a non-invasive method for CRC early diagnosis.

## Introduction

As one of the most common gastrointestinal tumors worldwide, colorectal cancer (CRC) ranks third in the world among men and second among women, affecting more than 1.36 million people every year (1). Most of the CRC patients display no symptoms at early stages; in addition, the majority of CRCs develop slowly from adenomatous precursors (2). It has been estimated that >95% of colorectal cancer (CRC) would benefit from curative surgery if diagnosed at earlier or intermediate stages (3–6). Thus, early detection is of vital importance for improving the survival of CRC patients. Conventional screening methods including barium enema, colonoscopy, and sigmoidoscopy are uncomfortable, invasive, time consuming and expensive (7, 8). Fecal occult-blood testing (FOBT) and serum carcinoembryonic antigen (CEA) test are non-invasive methods; however, they are compromised by its low specificity (9–13). More non-invasive screening methods with high specificity and high sensitivity should be established for early detection of CRC.

Massive efforts in whole-genome sequencing and genome-wide association studies show that genetic factors only explain a small proportion of disease variance (14), and only about 5% cancers occur in the setting of a known genetic predisposition syndrome (15). It has been established that epigenetic regulation altering gene expression alone or in combination with inherited or somatic mutation plays important contribution to CRC (16). As a result, an intensive effort has been undertaken on CRC early diagnosis, which largely focuses on the methylation detection of tumor DNA or combined with the mutation deletion on certain genes (17–19). More importantly, the epigenetic alteration can be strongly affected by some environmental aspect, including diet habits or chronic alcohol consumption, which also affect human gut microbiota (20).

The gut microbiota maintains survival and metabolism with nutrients in the human body and works with the human body to respond to external environmental factors, carry out metabolic and immune activities, and maintain human health (21). Studying the intestinal microbiome composition of colorectal cancer patients can open new inspection methods for tumor screening. Recent studies, including ours, have suggested that microbiota profiles determined by high-throughput sequencing may be effective in predicting CRCs (22). It has been reported that peptostreptococcus anaerobius, an anaerobic bacterium enriched in the fecal and mucosal microbiota from CRC patients, promotes CRC (23). In addition, a number of bacteria, including *Bacteroides fragilis* and a strain of *Escherichia coli* (24–29), *Streptococcus bovis* (30, 31), *Clostridium septicumand* (32), and *Fusobacterium nucleatum* (33, 34) have been reported to be associated with CRC. Furthermore, metagenomic analysis of fecal microbiome has been performed and a couple of gene markers have been identified and validated as biomarkers for early diagnosis of CRC (35). Difference in gut microbiota between colorectal cancer patients and healthy people combined with other methods such as fecal immunochemical test (FIT), CEA, or other risks factors such as age and BMI index is required for improving accuracy (22, 36).

We evaluated differences in bacterial communities in stool samples of colorectal cancers and non-cancer controls through 16S rRNA high-throughput sequencing. In addition, 12 microbial biomarkers combined with FOBT have been identified for non-invasive early diagnosis of CRC.

## Materials and Methods

### Study Participants and Stool Samples Collection

Stool samples were collected from 382 individuals undergoing colonoscopy at the endoscopy center of Liaoning Cancer Hospital and Dongfang Hospital Affiliated to Tongji University, including 226 CRCs and 156 healthy controls. To avoid potential alternation of the gut microbiota, the exclusion criteria were: (1) past history of any cancer; (2) use of antibiotics within the past 3 months; (3) had surgery or invasive procedure within the past 3 months; and (4) had an inflammatory bowel disease. All enrolled subjects were asked to keep a steady dietary schedule and lifestyle and leave fecal sample over 1.0 g in a special containment before bowel preparation for any endoscopy or surgery. After stool collection from the patients, samples were stored at −80°C immediately for further analysis.

### 16S rRNA Gene Sequencing

DNA from stool samples was extracted using Qiagen QIAamp DNA Stool Mini Kit (Qiagen) according to instructions of the manufacturer. Quality and quantity of extracted DNA were examined by electrophoretic separation in a 0.8% (wt/vol) agarose gel and NanoDrop 2000 spectrophotometer, respectively.

The hypervariable V3–V4 regions of the 16S rRNA gene were amplified using the primer set of 338F (5′-ACTCCTACGGGAGGCAGCA-3′) and 806R (5′-GGACTACHVGGGTWTCTAAT-3′). PCR amplification uses Pfu high-fidelity DNA polymerase from TransGen Biotech and strictly controls the number of amplification cycles to keep the number of cycles as low as possible while ensuring the same amplification conditions for the same batch of samples. PCR amplification, purification of amplified product, sequencing library preparation, and pyrosequencing were performed at paired-end 250 bp on the Illumina MiSeq platform by Personal Biotechnology, Co., Ltd. (Shanghai, China).

### Sequence Data Processing

Raw sequencing data were processed using Quantitative Insights into Microbial Ecology (QIIME) v1.8.0 (37) and filtered by removing tags and primers. A quality cut-off was applied to discarding the reads (1) that are shorter than 150 bp, with (2) an average Phred score lower than 20, (3) with ambiguous bases. After that, the filtered reads were assembled using FLASH software v1.2.7 with overlapping between the paired-end reads >10. Chimeric sequences were filtered using USEARCH v5.2.236 (http://www.drive5.com/usearch/). After quality filtering and chimera removal, clean reads were then clustered into operational taxonomic units (OTUs) at 97% sequence identity using UCLUST. The taxonomic classification was performed with Greengenes database release 13.8. Alpha diversity indices of Chao1, ACE, Simpson and Shannon were estimated. Beta diversity analysis was performed with UniFrac in QIIME. Non-metric multi-dimensional scaling (NMDS) was generated by R language release package for analysis based on distance.

### Fecal Occult Blood Test

All enrolled subjects were asked to offer a valid fecal occult blood test report from a community hospital or a general hospital in recent 6 months. Stool samples with blank FIT result would have to be examined using Fecal Occult Blood Diagnostic Kit (Colloidal Gold) (Chemtrue^@^) which had been approved by the Chinese Food and Drug Administration Bureau. The cut-off value for positive FOBT was 200 ng/ml according to the instructions of the manufacturer.

### Statistical Analysis

Significant differences among treatments were identified through one-way analysis of variance (ANOVA) followed by Tukey’s test. Typically, homogeneity of variance for the obtained data was tested, and data of the test values >0.05 were adopted for the ANOVA analysis. All statistical analyses were performed using SPSS 19.0 (IBM, New York, USA), and significant levels were reported at p <0.05 and p <0.01.

FOBT test results were recorded as positive or negative.

### Classifier Construction

We used an SVM (support vector machine) (R 3.6.1; the e1071 R package) to build the classifier for colorectal cancer with genera abundances as features. All the genera were normalized, and rare genera with less than 20% occurrences in all samples were removed. To filter out redundant features from the resulting 107 genera, the mRMR algorithm (38) was performed to 10 data sets with 50 features, each using the R package “mRMRe”. Leave-one-out cross-validation LDA (linear discriminant analysis) was applied to determine how many features to be used. We tuned both radial basis function (RBF) kernel (Gaussian kernel) and a linear kernel function of SVM with a tolerance of 0.001 to get a better performance using 10-fold cross-validation. For RBF kernel, the penalty parameter C was varied as {1e-4, 1e-3…, 1e4} and the gamma parameter G as {1e-5, 1e-3…, 1e3}. For linear kernel, the penalty parameter C was varied as {1e-4, 1e-3…, 1e4}. FOBT test from stool sample has been widely used in diagnosis; genera data together with FOBT test result were selected as feature set as well. Matthews correlation coefficient (MCC) was chosen as indicator of the performance. Receiver operating characteristic (ROC) figures were drawn using R package “pROC”.

## Results

### The Gut Microbiome Is Dysbiotic in Colorectal Adenocarcinoma Patients

After quality filtering and primer trimming, a total of 5,153 usable high-quality sequence reads were generated from 382 samples, the length of which was about 468 bp. In this study, a total of 4,728 OTUs were obtained from the colon cancer group and 4,331 OTUs from the healthy control group. A total of 3,906 OTUs were shared among the two groups. Compared with 423 unique OTUs from the healthy group, CRC group contained 822 unique OTUs (**Supplemental Figure S1**). Rarefaction curves of CRC and control samples almost plateaued, suggesting the sequencing was sufficient (**Supplemental Figure S2**).

**Figure 1:**
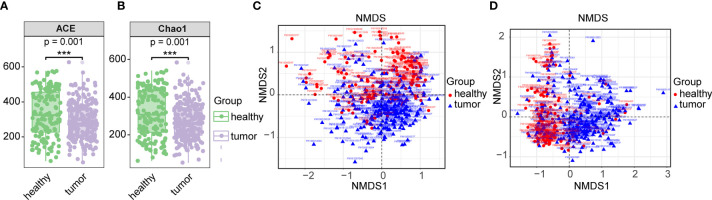
Comparison of gut microbiome between CRC and healthy gut. **(A)** Alpha diversity communities based on observed OTUs by richness (Chao1, ACE). **(B)** Beta diversity measured by unweighted unifrac. **(C)** Beta diversity measured by unweighted unifrac. **(D)** Beta diversity measured by weighted unifrac. Overall differences in the microbiome composition among groups were assessed by ANOSIM. *** means biological significance.

Based on the total OTU statistical sequence, fecal microbial richness, as estimated by ACE and Chao1 (P-values <0.001, respectively), was significantly decreased in CRC (**Figures 1A, B**). The fecal microbial diversity, estimated by Shannon and Simpson, did not show significance between control and CRCs (**Supplemental Figure S3**). When microbiota composition between CRC and healthy gut was compared, beta-diversity exhibited difference between two groups (p = 0.001) (**Figures 1C, D**). These results suggested dysbiosis in the gut microbiome of CRC patients.

### The Divergent Taxonomic Composition and Functional Performance of Microbiota in CRC and Healthy Gut

After quality filtering, sequences at a 97% sequence similarity were selected for taxonomic composition analysis; 21 bacterial phyla, 34 microbial class, 56 microbial orders, 107 microbial families, 209 microbial genera, and 268 microbial species have been identified **(** **Supplemental Table S1**).

**Figure 2:**
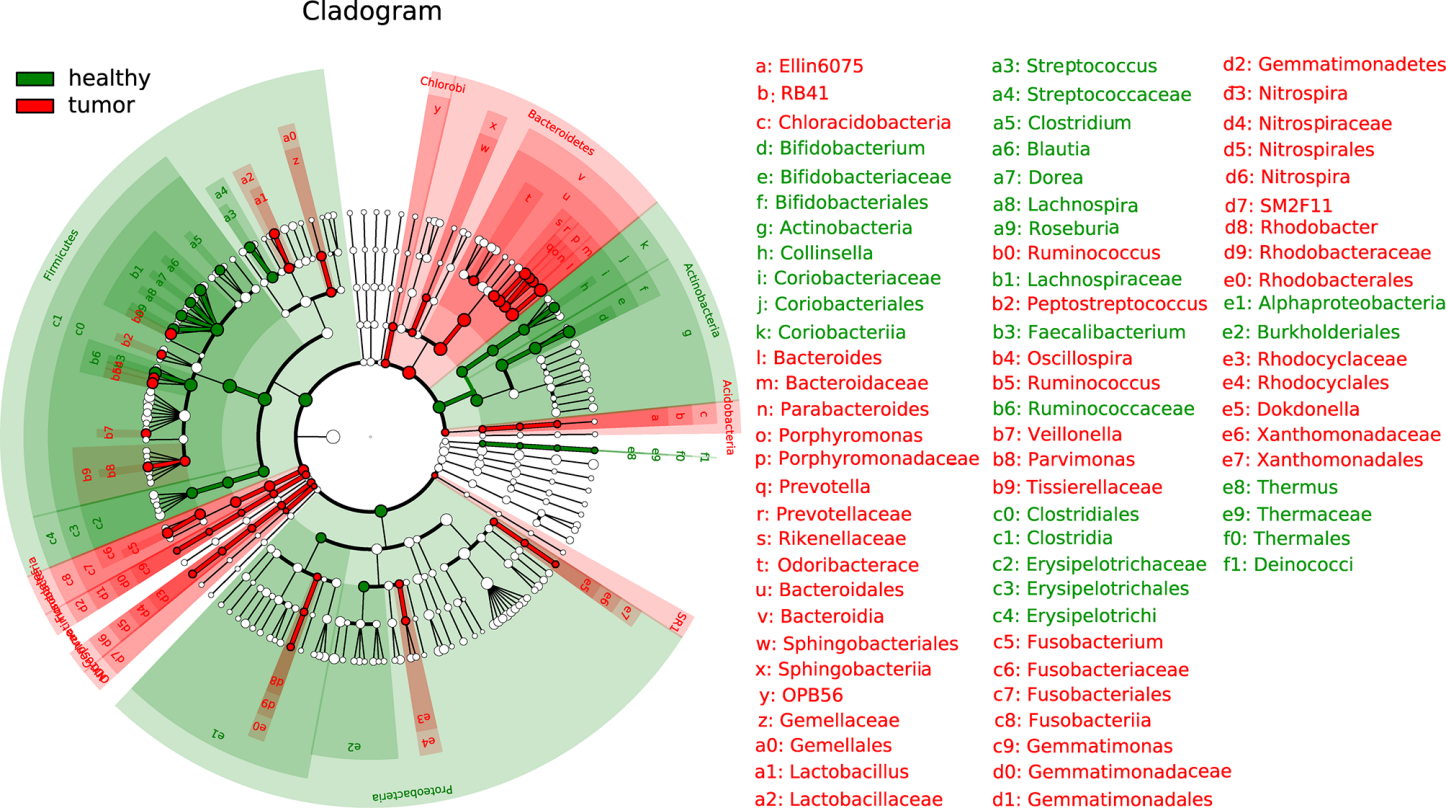
Linear discriminant analysis effect size (LEfSe) analysis of gut microbiota composition between two groups.

The LEfSe (linear discriminant analysis effect size) analysis was performed to determine differences in bacterial taxonomy. The histogram with cladogram showed that overall phylum Bacteroidetes was highly accumulated in CRC while overall phylum Actinobacteria was overall less accumulated in health samples. Divergent alteration was observed at lower taxonomic levels from phylum Firmicutes and Proteobacteria (**Figure 2**).

**Figure 3:**
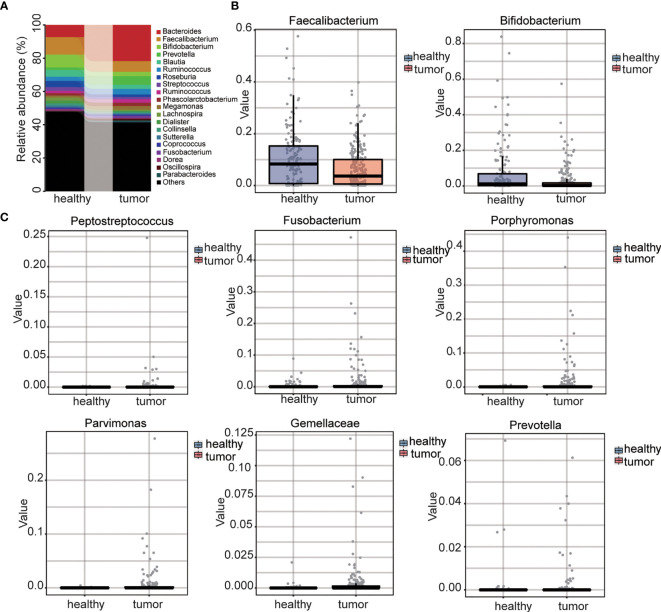
Analysis of genus level and selected genus between CRC and control. **(A)** The distribution of two groups at genus level. **(B)** Abundance of benefit intestinal bacteria between two groups. **(C)** Abundance of CRC related intestinal bacteria between two groups.

We further compared the difference between control and CRC microbiome at different levels. At the phylum level, four phyla were detected with relatively high abundance. CRC samples showed increased abundance of phylum Bacteroidetes while healthy samples showed increased abundance of Firmicutes, Proteobacteria, and Actinobacteria (**Supplemental Figure S4A**). At the family level, for the family with relatively high abundance, CRC samples had higher abundances of Bacteroidaceae, Veillonellaceae and Prevotellaceae whereas healthy samples had higher abundances of Ruminococcaceae, Lachnospiraceae, Enterobacteriaceae, and Bifidobacteriaceae (**Supplemental Figure S4B**). At the genus level, the most abundant genera identified in healthy samples were *Faecalibacterium* (10.49%), *Bifidobacterium* (7.65%), and *Bacteroides* (7.33%), while in CRC gut, the most abundant genera were *Faecalibacterium* (6.37%), *Bacteroides* (21.79%), and *Prevotella* (5.14%) (**Figure 3A**). The most abundant gut microbe, *Fecalibacterium*, termed as the marker of healthy gut (39), decreased by 60%. Beneficial intestinal bacteria Bifidobacterium (40) decreased by 35% in the CRC gut (**Figure 3B**). Importantly, the role of several genera the role in CRC, such as *Peptostreptococcus* (23), *Fusobacterium* (33, 34), *Porphyromonas*, *Parvimonas*, *Gemella*, and *Prevotella* (41) had been reported; they were extremely upregulated in the CRC gut (**Figure 3C** and **Supplemental Table S2**). The abundance of all gut microbes was listed as **Supplemental Table S1**. These results strongly suggested the dysbiosis in the CRC gut.

**Figure 4:**
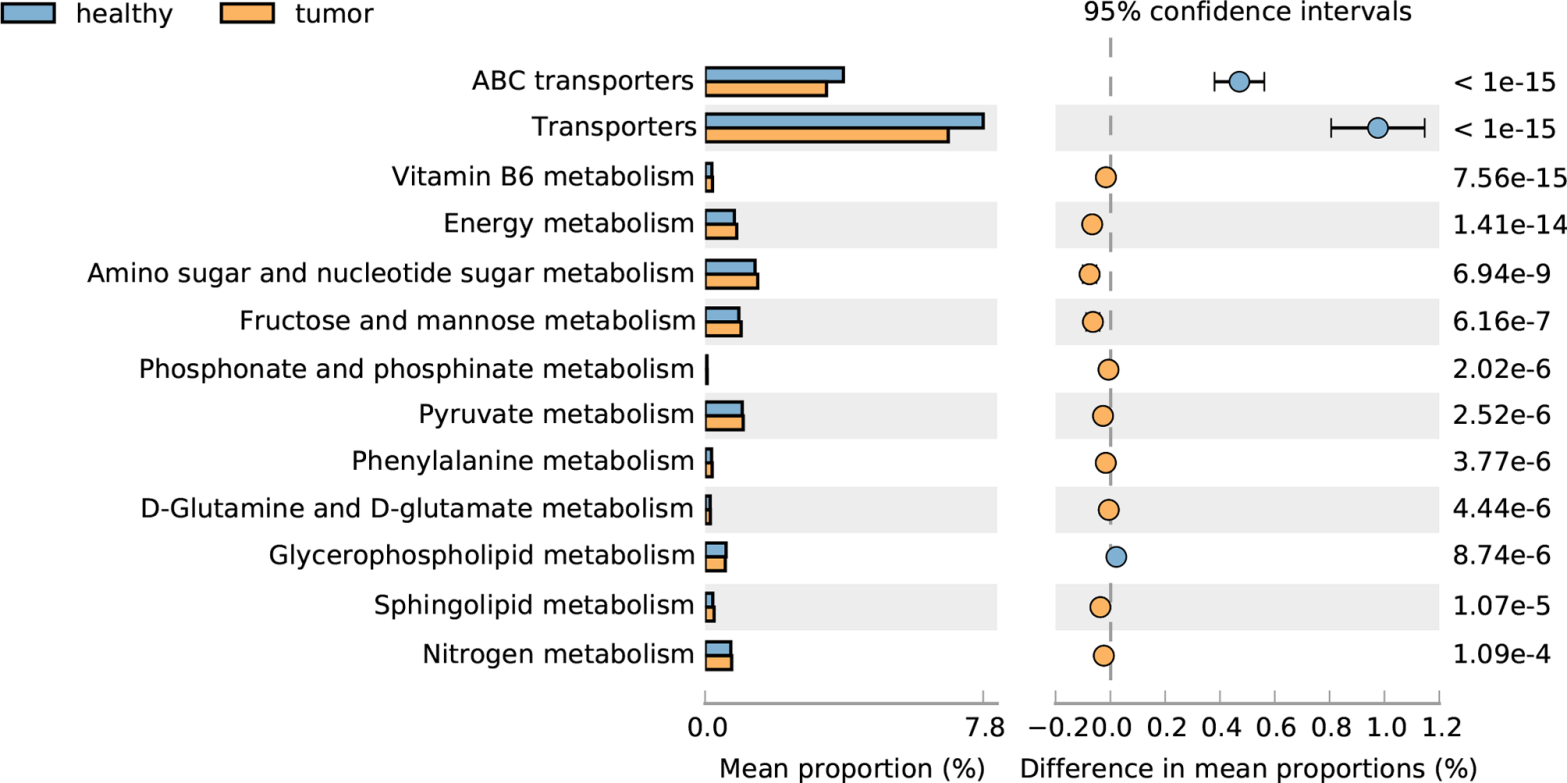
Pathways altered in CRC and healthy guts.

We further compared the functional capacity of the gut microbiota between CRC and healthy subjects; the transporter pathway, especially the ABC transporter pathway, is significantly increased in CRC gut, and a large number of metabolism related pathways, such as vitamin B6 metabolism, energy metabolism, amino sugar and nucleotide sugar metabolism, fructose and mannose metabolism, phosphonate and phosphinate metabolism, pyruvate metabolism, phenylalanine metabolism, D-Glutamine and D-glutamate metabolism, sphingolipid metabolism, and nitrogen metabolism decreased in CRC gut, with the exception of glycerophospholipid metabolism. These results suggested the disorder of metabolism in CRC patients (**Figure 4**).

### Fecal Microbial Biomarkers for Colorectal Adenocarcinoma Non-Invasive Diagnosis

The changes in the bacterial community between the two groups could be screened as biomarkers for colorectal cancer detection to assist in its diagnosis. To select the most relevant feature which could be term as biomarkers for CRC, the ExtraTrees classifier calculating feature importance score was performed. Forty significantly different features showing different abundances were selected for further analysis (**Supplemental Table S3**).

### Machine Learning Classification for CRC to Identify Fecal Microbial Biomarkers for Non-Invasive Diagnosis

**Figure 5:**
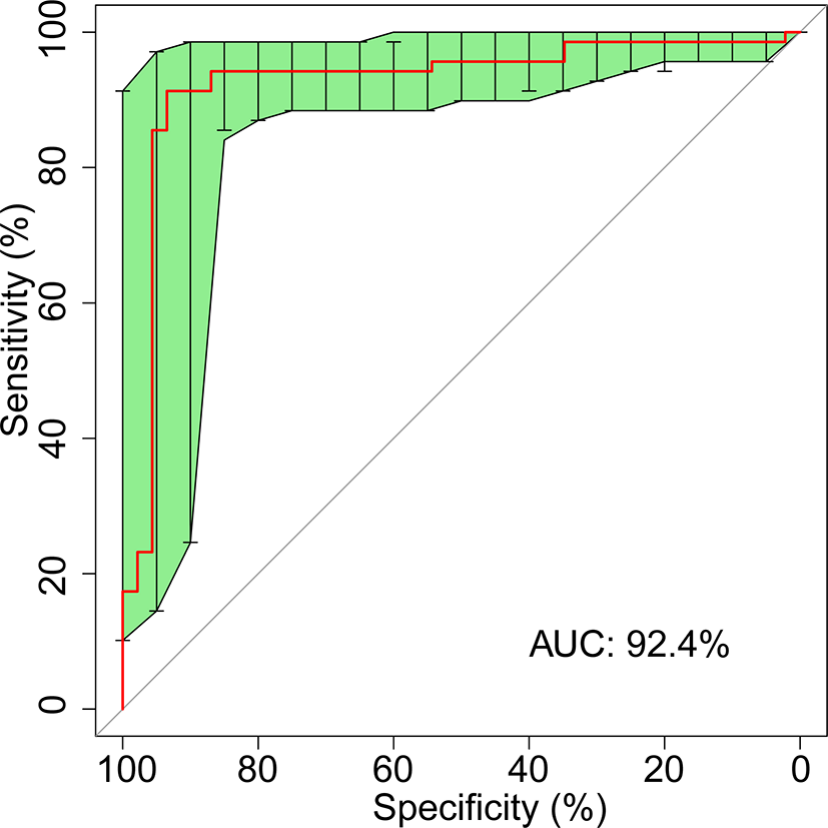
ROC curve for 12 fecal microbial markers plus FOBT.

To illustrate the diagnostic value of the selected biomarkers in the gut microbiome for colorectal cancer and find out a smaller number of biomarkers for diagnosis, we constructed a classifier established by SVM (Support Vector Machine) model to detect cancerous samples. We selected 267 samples as training set, including 141 from Liaoning and 126 from Shanghai; in addition, the rest 115 samples were selected as verification set, including 61 from Liaoning and 54 from Shanghai. The SVM model used 13 genera (**Supplemental Table S4**) from bacteria as features to distinguish CRC patients from healthy controls with a sensitivity of 82.6%, specificity of 78.3%, precision of 85.1%, and accuracy of 80.9% under the para1 conditions (‘C’: 1,000, ‘gamma’: 1e-5, ‘kernel’: ‘radial’) (**Supplemental Table S5** and **Supplemental Figure S5**). We also constructed the classifier from bacteria together with FOBT; 13 features including 12 bacteria genera and FOBT (**Table 1**) could distinguish CRC and healthy people. The sensitivity, specificity, precision, and accuracy increased into 91.3, 93.5, 95.4, and 92.2% under the para2 conditions (‘C’: 10, ‘kernel’: ‘linear’) (**Table 2** and **Figure 5** **)**. The 12 fecal bacteria and FOBT could be termed as non-invasive biomarkers for colorectal adenocarcinoma.

**Table 1:** The weight for CRC and healthy gut classification of 12 bacteria genus and FOBT.

Parvimonas
Parabacteroides
Clostridium
Odoribacter
SMB53
Dorea
Prevotella
Blautia
Lachnospira
Roseburia
Bacteroides
FOBT
Faecalibacterium

**Table 2:** The performance of 13 biomarkers including bacteria genus and FOBT.

	Sensitivity	Specificity	Precision	Accuracy	TP	FP	TN	FN
Bacteria genus with FOBT	91.3%	93.5%	95.4%	92.2%	63	3	43	6

## Discussion

CRC is a high risk cancer in China and all over the world. Early detection and treatment of CRC are important for improving the late survival rate and reducing the cost of late treatment. Colonoscopy and FOBT are widely used in CRC screening; however, for their low compliance or sensitivity, more non-invasive and painless methods with high sensitivity and specificity are required (7–13). Circulating tumor DNA (ctDNA) is an extracellular DNA that originates from tumor cells and circulates in several bodily fluids, including blood, synovial fluid, and cerebrospinal fluid (42). For the similarity of genetic and epigenetic information provided by ctDNA to that of invasive tumor biopsies, ctDNA has been widely used to detect the gene mutation and is termed as a non-invasive diagnostic tool for several cancers (43). In many tumors, increased methylation of tumor suppressor genes occurs at an early stage, thus, ctDNA methylation profiling detection can be used as an alternative non-invasive diagnostic tool (44–46). Some specific DNA methylation sites, such as SEPT9, have been identified as biomarkers of CRC (47, 48). However, the extremely low level in the blood and the non-organ information of ctDNA present a great challenge to early diagnosis.

The gut microbiome plays a major role in protecting the host against the overgrowth of pathogens and in sustaining the health of colon. There is intensive evidence revealing the close relationship between gut microbiome and colorectal cancer (49–51). In clinical application, changes of gut microbiome can be regularly monitored, thus, revealing gut microbiome divergence between CRC and healthy control would help to find out the weighted bacteria for distinguishing them, and the bacteria can be termed biomarkers for CRC early diagnosis.

We have performed high-throughput sequencing on the v3–v4 regions of intestinal bacteria 16S rRNA gene in stool and described the patterns of gut microbiome relative to healthy control and CRC patients. Fecal richness from colorectal cancer patients decreased; in addition, the proportion of various beneficial bacteria decreased, and the proportion of harmful bacteria significantly increased. A dozen of opportunistic pathogens including *Bacteroides* and *Prevotella* were significantly increased in patients with colorectal cancer (**Figure 3**). A couple of pathogens, including *Fusobacterium nucleatum* (33, 34), *Peptostreptococcus anaerobius*, and enterotoxigenic *Bacteroides fragilis* (52), whose roles have been established in CRC induction, were highly accumulated in CRC patients (**Figure 3** and **Supplemental Table S1**). The identification of dysbiosis characteristics could be facilitated, and they can be considered as taxonomic biomarkers for CRC screening.

We performed machine learning using SVM model between pairs of cohorts to conduct binary classification for classifying CRC patients and control. A variety of features including taxonomic, functional (53), and k-mer-based (54) classification schemes have been used for machine learning approaches. Here, we used 13 genera of the gut bacteria to show their great contribution in differentiating the CRC state *versus* control for machine learning. In addition, FOBT test results were selected as a their importance on CRC diagnosis in clinical practice. Our machine learning results showed high performances in CRC *versus* control models (**Table 1** and **Figure 5**). *Parvimonas*, *Prevotella* (41), *Clostridium* (32), *Dorea* (55), and *Bacteroides* (56) have been reported to have higher accumulation in CRC gut. On the other side, *Blautia* and *Faecalibacterium* have been reported to have lower accumulation in CRC gut (57). Notably, Blautia, Lachnospira, and Roseburia belong to Lachnospiraceae, whose lower levels were associated with CRC (58). In our results, the genera beneficial for health were increased, and the genera harmful for health were reduced in the CRC gut. In addition, the association of Odoribacter and SMB53 with CRC was reported for the first time. The high performance of fecal bacteria and FOBT test from stool sample facilitates the establishment of a new non-invasive method for an examination of colorectal cancer.

In addition to causing intestinal diseases, gut microbiome also contributes to obesity, diabetes, allergic asthma, and neuropsychiatric diseases (59–61); thus, clinical monitoring of fecal bacteria can assist in the diagnosis of other diseases related to gut microbiome. Furthermore, gut status could be improved by artificially guiding the intervention of diet or the intake of beneficial bacteria according to the changes of gut microbiome (62), and the improvement could be easily detected from the fecal bacteria. Thus, gut microbiome has become a hot spot in clinical research.

In summary, we monitored the gut microbiome and took 12 bacteria genus and FOBT displaying high weight for classification between CRC and healthy gut as biomarkers for CRC early diagnosis. The method benefits those who cannot receive colonoscopy in a short time and those who are not willing to use colonoscopy. Compared with the existing methods of CRC diagnosis, our method is non-invasive and painless; not only does it not require complex examination and preparation before sampling, but also improves the sensitivity and specificity of the test compared with the FOBT alone.

## Data Availability Statement

The original contributions presented in the study are publicly available. This data can be found here: NCBI repository, accession number: PRJNA706061.

## Ethics Statement 

The studies involving human participants were reviewed and approved by Ethics Committee of Liaoning Cancer Hospital and Dongfang Hospital Affiliated to Tongji University. The patients/participants provided their written informed consent to participate in this study.

## Author Contributions

BY and BM prepared the samples, extracted the DNA for 16s rRNA sequencing and revised the manuscript. JY analyzed the 16s rRNA sequencing data and wrote the manuscript. QM helped to prepare the samples. TD helped to perform the data analysis and organize the data. HL and YZ performed the machine learning analysis. ZS revised the manuscript. SM and CS designed and leaded the project. All authors contributed to the article and approved the submitted version.

## Funding

This study was supported by Innovation Fund of Science and Technology Committee in Shanghai Pudong New Area (Nos. PKJ2016-Y60), National Natural Science Foundation of China (Nos. 81871953) and Jiangxi Youth Science Fund (Nos. 20171BAB215043) National Science Foundation of China, Grant/Award Number: 81902383, Revitalizing Liaoning Talents Program, Grant/Award Number: XLYC1907004

## Conflict of Interest

JY, YZ, ZS and SM were employed by Shanghai Personal Biotechnology Co., Ltd.

The remaining authors declare that the research was conducted in the absence of any commercial or financial relationships that could be construed as a potential conflict of interest.

## Publisher’s Note

All claims expressed in this article are solely those of the authors and do not necessarily represent those of their affiliated organizations, or those of the publisher, the editors and the reviewers. Any product that may be evaluated in this article, or claim that may be made by its manufacturer, is not guaranteed or endorsed by the publisher.

## Supplementary Material

Supplementary Figure 1 Venn diagram based on OTU. Each ellipse represents a group of samples. The overlapping area between the ellipses represents the shared OTU among the sample groups, and number of each block indicates the number of common or unique OTU of the sample groups contained in the block.

Supplementary Figure 2 Rarefaction curves of CRC and control samples.

Supplementary Figure 3 Alpha diversity communities based on observed OTUs by diversity (Simpson, Shannon).

Supplementary Figure 4 The distribution of two groups at phylum level **(A)** and family level **(B)**.
