# Supplementary figures and images for: Fecal Bacteria as Non-Invasive Biomarkers for Colorectal Adenocarcinoma
Source: Front Oncol. 2021 Aug 10;11:664321. doi: 10.3389/fonc.2021.664321 (PMC8383742; doi:10.3389/fonc.2021.664321)

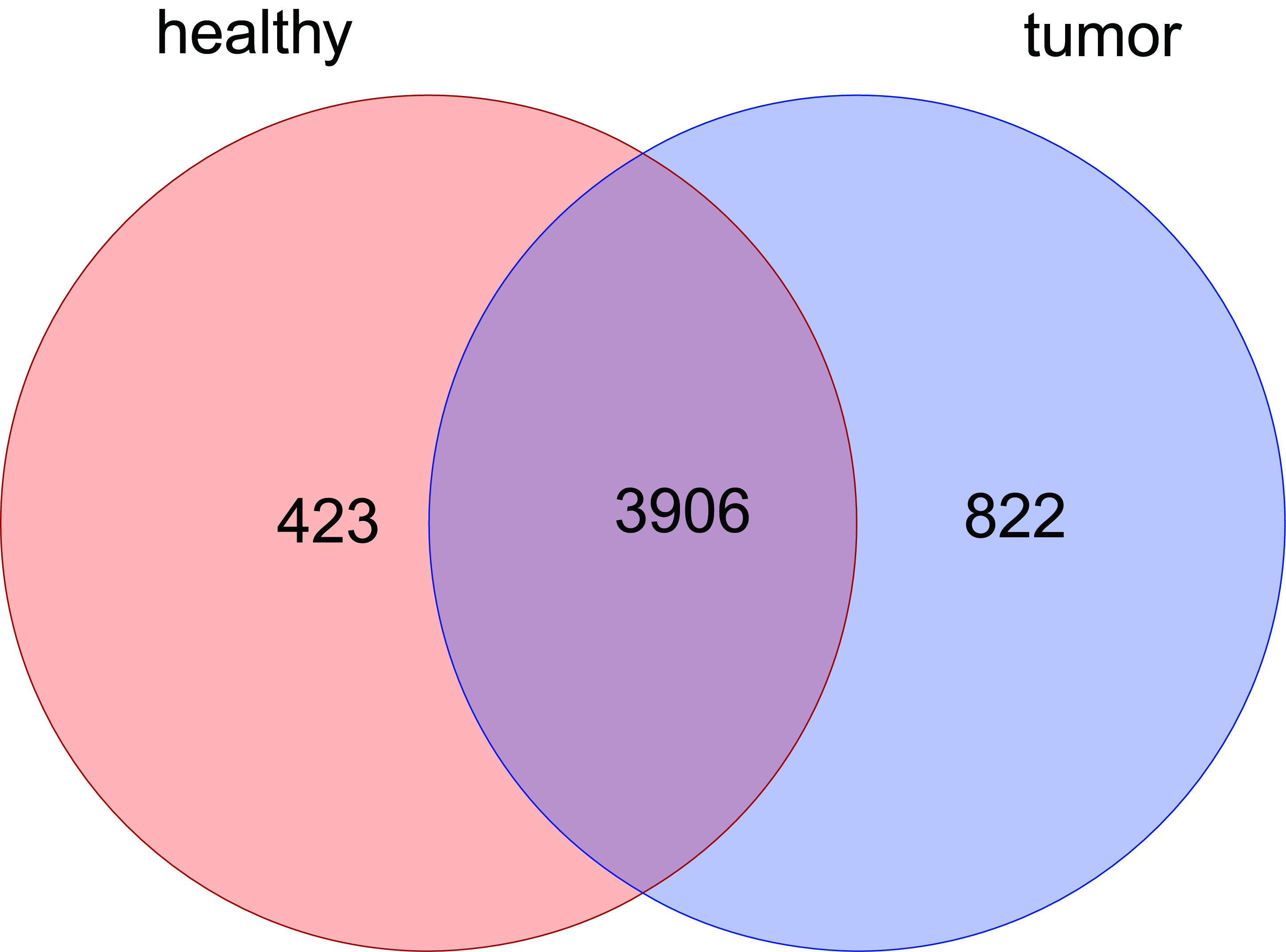

Supplement: Supplementary Figure 1 — Venn diagram based on OTU. Each ellipse represents a group of samples. The overlapping area between the ellipses represents the shared OTU among the sample groups, and number of each block indicates the number of common or unique OTU of the sample groups contained in the block. [file Image_1.tif]

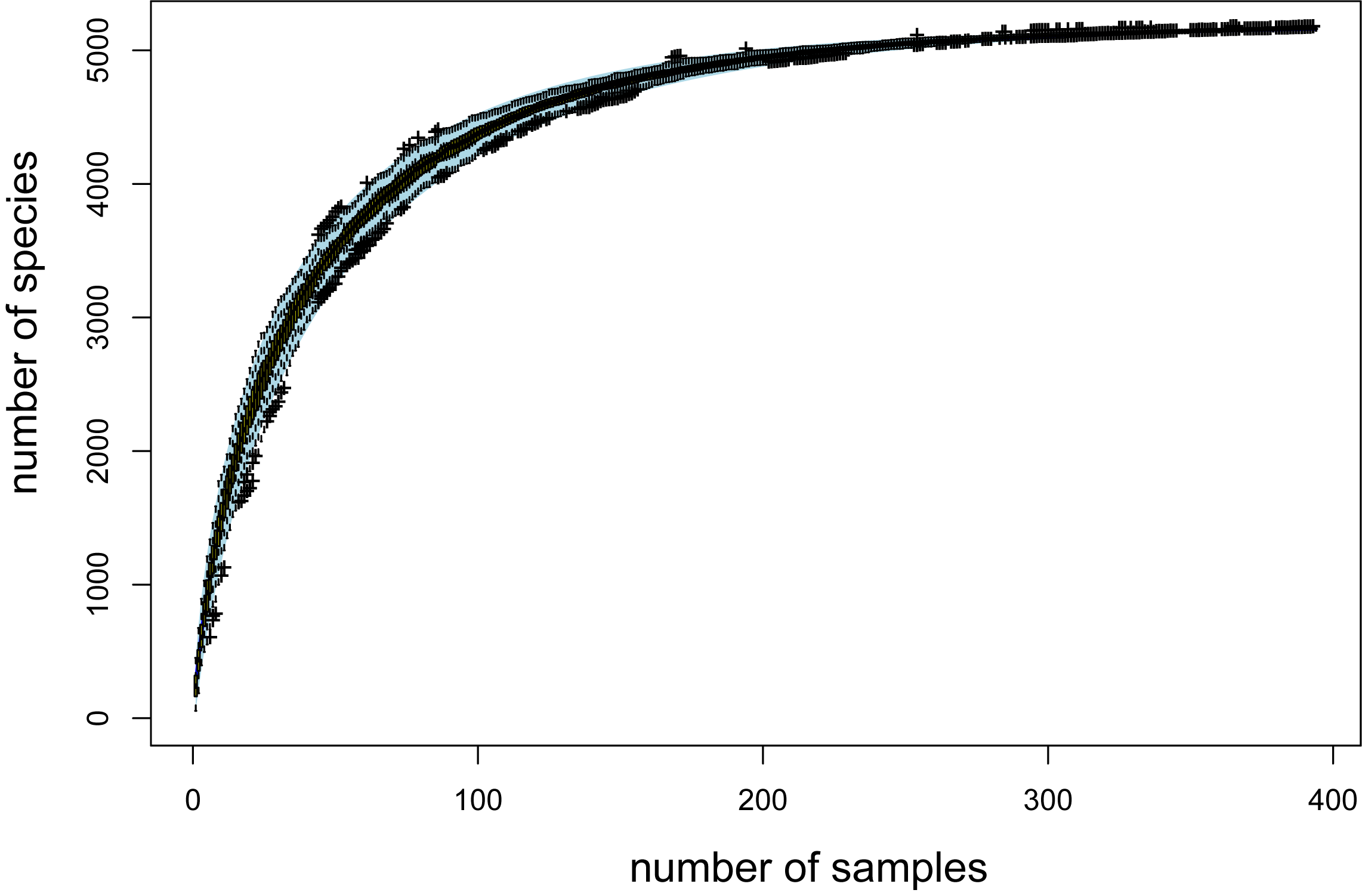

Supplement: Supplementary Figure 2 — Rarefaction curves of CRC and control samples. [file Image_2.tif]

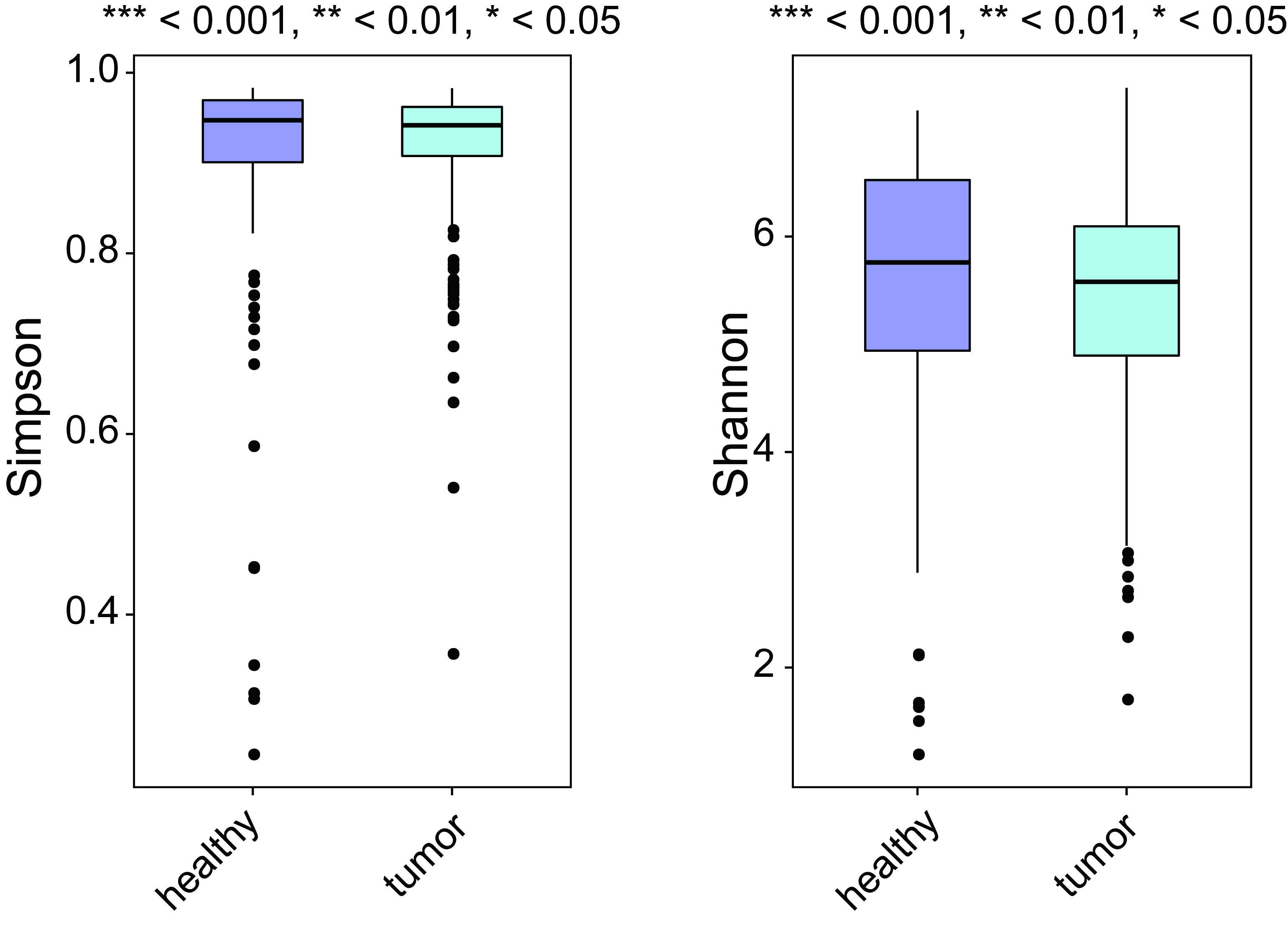

Supplement: Supplementary Figure 3 — Alpha diversity communities based on observed OTUs by diversity (Simpson, Shannon). [file Image_3.tif]

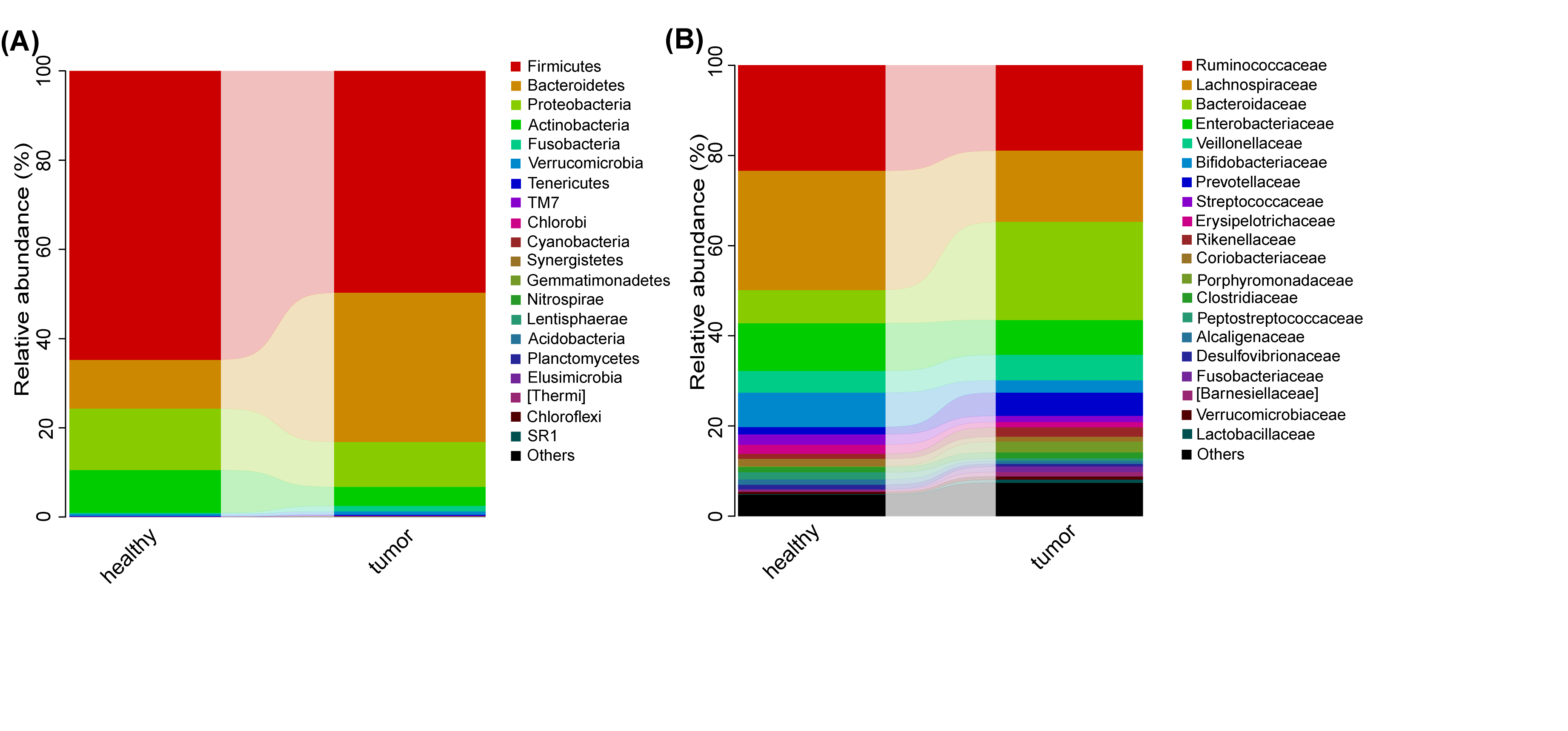

Supplement: Supplementary Figure 4 — The distribution of two groups at phylum level (A) and family level (B). [file Image_4.tif]

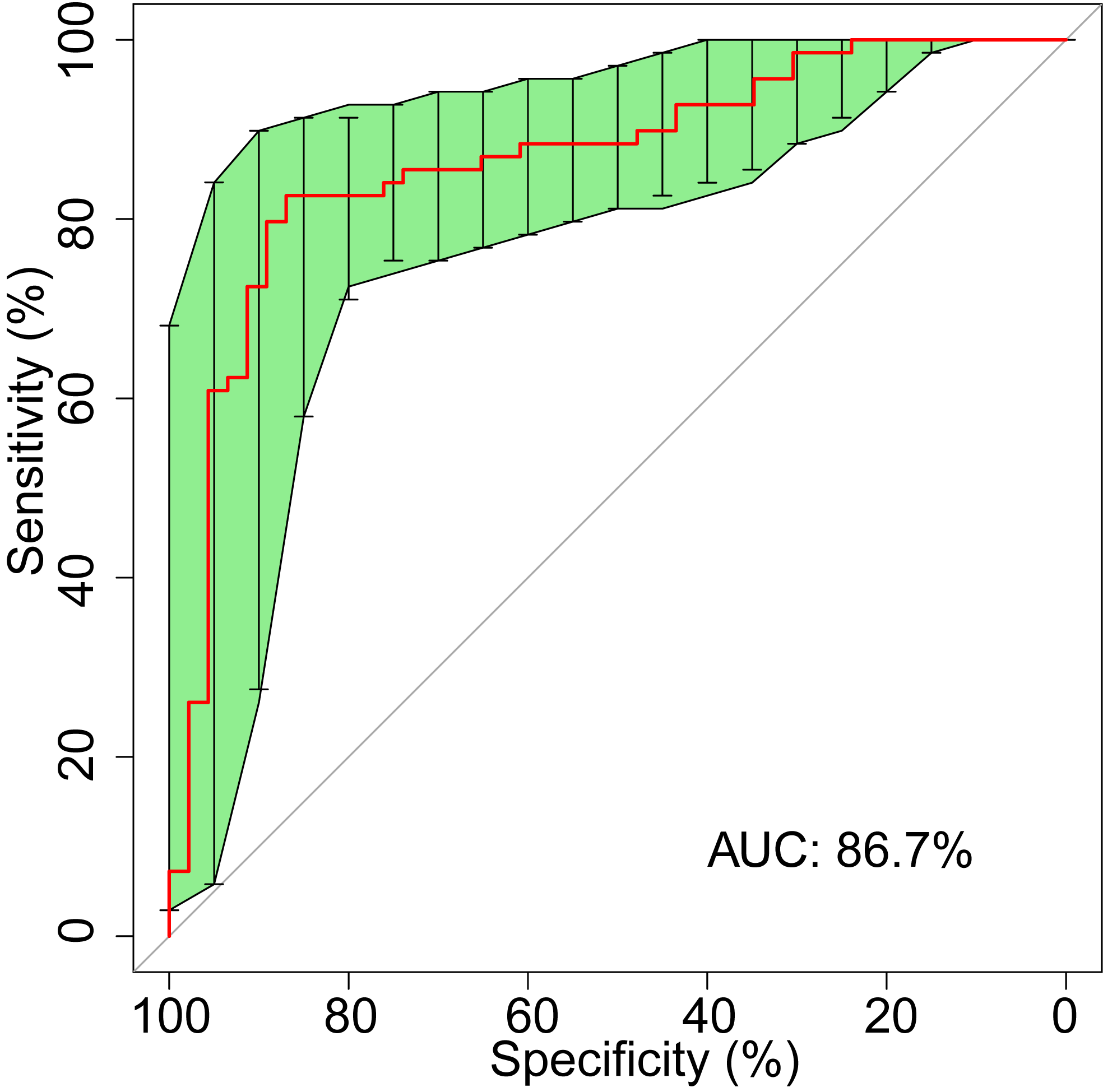

Supplement: Supplementary file 5 [file Image_5.tif]
